# Raising standards in clinical research – The impact of the ECRIN data centre certification programme, 2011–2016

**DOI:** 10.1016/j.conctc.2017.02.005

**Published:** 2017-02-14

**Authors:** C. Ohmann, S. Canham, J. Demotes, G. Chêne, J. Lauritsen, H. Martins, R.V. Mendes, E.B. Nicolis, A. Svobodnik, F. Torres

**Affiliations:** a Chair of ECRIN Independent Certification Board and Network Committee, ECRIN, Düsseldorf, Germany; b Scientific Secretary of ECRIN Independent Certification Board, ECRIN, Surrey, UK; c Director-General of ECRIN, ECRIN, Paris, France; d Member of ECRIN Independent Certification Board, Centre d’Investigation Clinique-Epidémiologie Clinique, Bordeaux, France; e Member of ECRIN Independent Certification Board, Department of Clinical Medicine, Odense University Hospital, Odense, Denmark; f Member of ECRIN Independent Certification Board, Serviços Partilhados do Ministério da Saúde, Lisboa, Portugal; g Member of ECRIN Independent Certification Board, Shared Services of Ministry of Health, Lisboa, Portugal; h Member of ECRIN Independent Certification Board, Cardiovascular Research, Clinical Drug Evaluation, Mario Negri Institute for Pharmacological Research, Milano, Italy; i Member of ECRIN Independent Certification Board, St. Ann’s University Hospital, Brno, Czechia; j Member of ECRIN Independent Certification Board, Medical Statistics Core Facility, IDIBAPS, Hospital Clinic Barcelona, Barcelona, Spain

## Abstract

The nature and the purpose of the ECRIN Data Centre Certification Programme are summarised, and a very brief description is given of the underlying standards (129 in total, divided into 19 separate lists). The certification activity performed so far is described. In a pilot phase 2 centres were certified in 2012. Calls in 2014 and 2015 resulted in a further 8 certified centres, with 2 certifications still in progress, and the 2016 call has generated several additional applications. The impact and benefits of the programme are listed, divided into a) the effects of the introduction of the standards, b) the effects of the certification programme in general, and c) the effects of the certification programme on individual units. The discussion emphasises the generally positive impact of the programme so far but stresses the need to better clarify the perspective and role of the programme.

## 1 Introduction

The European Clinical Research Infrastructure Network, ECRIN, is a non-profit organisation which aims to facilitate multi-national clinical research across Europe. It does that by providing a wide variety of tools and services, as well as informational support, and by implementing programmes promoting greater consistency and quality in clinical trial management [1].

Since 2013 ECRIN has been one of several ‘ERIC's, i.e. a European Research Infrastructure Consortium, funded by direct contributions from the governments of the ECRIN member countries (which are not restricted to member states of the EU – the European Union). ECRIN first developed, however, as an infrastructure project within the EU's centrally funded research programmes, Framework Programmes (FP) 6 and 7.

In 2009, within FP7, an ECRIN workgroup charged with developing support for data management within clinical trials units carried out a survey of information technology (IT) and data management practices in non-commercial trial units across Europe. This confirmed the anecdotal evidence, of considerable heterogeneity in both systems and practices. Many different software products for data management were being used in clinical trials units, and these included many proprietary, locally developed solutions rather than professional products [2]. Deficits in the quality of data management were also observed in some units, including missing SOPs (standard operating procedures) and limited validation and change management, of both underlying systems and individual trial databases.

Most academic centres are constrained by limited human and financial resources and, historically, had sometimes found it difficult to provide levels of IT and data management which were fully and formally compliant with Good Clinical Practice (GCP), especially in the context of complex international trials. With the stimulus of the 2001 European Directive [3] and the consequent national legislation, academic trials units were certainly developing their quality management systems, including in data management. But one crucial problem was that there was no public, freely available and widely accepted interpretation of what GCP compliance meant for IT and data management in practical, concrete terms.

For that reason ECRIN decided, in the framework of the FP7 ECRIN project, to develop a set of quality standards or requirements. Taken together these would describe GCP-compliant IT and data management, with the emphasis on pragmatic measures that were feasible for non-commercial units. The standards were to be freely available, and were intended to be used for, amongst other things, quality management, validation, preparation of audits, and staff training.

Furthermore, ECRIN would use these requirements as a basis for selection and certification of recognised ‘ECRIN data centres’, units that could demonstrate compliance with the ECRIN standards and that could therefore be used with confidence within ECRIN supported programmes. As a consequence, after the first version of the standards were developed from 2009 to 2011, the ECRIN data centre certification programme was launched in 2011 (initially as a pilot), and it has been successfully implemented though yearly calls (2014, 2015, 2016).

The aims of the certification programme, and the standards that support it, have therefore always included the two specific aspects below, within the more general goal of explicitly setting out data management standards for clinical research:

a) to audit individual units against the standards, to confirm their ability to provide compliant, effective and efficient data management services for controlled clinical trials, and for ECRIN-supported multi-national trials in particular,
b) to provide a clear interpretation of regulatory and good practice requirements, in the particular context of non-commercial trials units in Europe, and so act as a practical guide to establishing and managing high-quality data management services.

In this paper we first summarise the methods used to bring about those aims, with an outline of both the standards and the ECRIN data certification programme to date. The results of the certification programme are then described, and the weaknesses and strengths of the programme discussed.

## 2 Methods

### 2.1 Developing the ECRIN data centre standards

The initial version of the standards were assembled by ECRIN domain experts from a wide variety of trials units and countries, using both face to face meetings and a modified Delphi process. The starting point was GCP, but a variety of international, European and national regulations and guidelines were also considered, discussed, distilled, adopted and adapted. These included:

• EU Directives for the implementation of GCP: 2001/20/EC, EU Directive 2005/28/EC, EU Directive 95/46/EC
• Computerized Systems, EudraLex - Volume 4, Annex 11
• EMEA Reflection paper on expectations for electronic source documents used in clinical trials
• Good practice for computerized systems in regulated GXP environments, PIC/S Inspectors Guide
• Good Automated Manufacturing Practice (GAMP^R^) Version 5 of the International Society for Pharmaceutical Engineering,
• FDA: Guidance for Industry: Computerized Systems Used in Clinical Trials and 21 CFR Part 11
• ISO 27001 Information Security Management - Specification.

These documents stimulated discussion and ideas, and sometimes directly suggested a requirement statement. But in general the ECRIN working group concentrated on defining standard requirements that, whilst certainly in line with the documents listed above, and with the principles of GCP in particular, reflected the special needs of data centres in non-commercial clinical trials units. Version 1.0 of the standards was produced in 2010. The process of creating them is described in more detail in Ref. [4].

In fact the 2011 pilot certification programme showed that the original requirements were too numerous and over complicated, being divided into both ‘essential’ and ‘best practice’ standards. The list was substantially simplified in 2012, with only the ‘essential’ standards retained. The revision process, also carried out by a group of ECRIN domain experts, and the 139 standards that remained after it, are described in Ref. [5]. The other significant change post-pilot was the introduction of paragraphs of explanatory material for each standard: to try and ensure a consistent interpretation of the requirements, to give examples and further practical guidance, and to indicate the evidence that would normally be expected to support a claim to compliance.

Feedback from the audits in 2014 generated a further review process in 2015, but this time the version that emerged (v3.0) exhibited a relatively small level of consolidation, with the 139 standards becoming 129. Much of the explanatory material was extended, however, because of:

a) A variety of requests for further clarification and guidance, for instance about system validation, and
b) The increased use by trials units of distributed IT infrastructures, e.g. the growing use of externally hosted database systems. It was necessary to clarify how the standards could be applied to both externally and locally hosted services.

Version 3.0 of the standards can be downloaded at [6]. Further reviews are likely in the future but we hope changes in the standards themselves will be relatively minor, though the explanatory material may continue to expand.

### 2.2 The current ECRIN standards

**Fig. 1:**
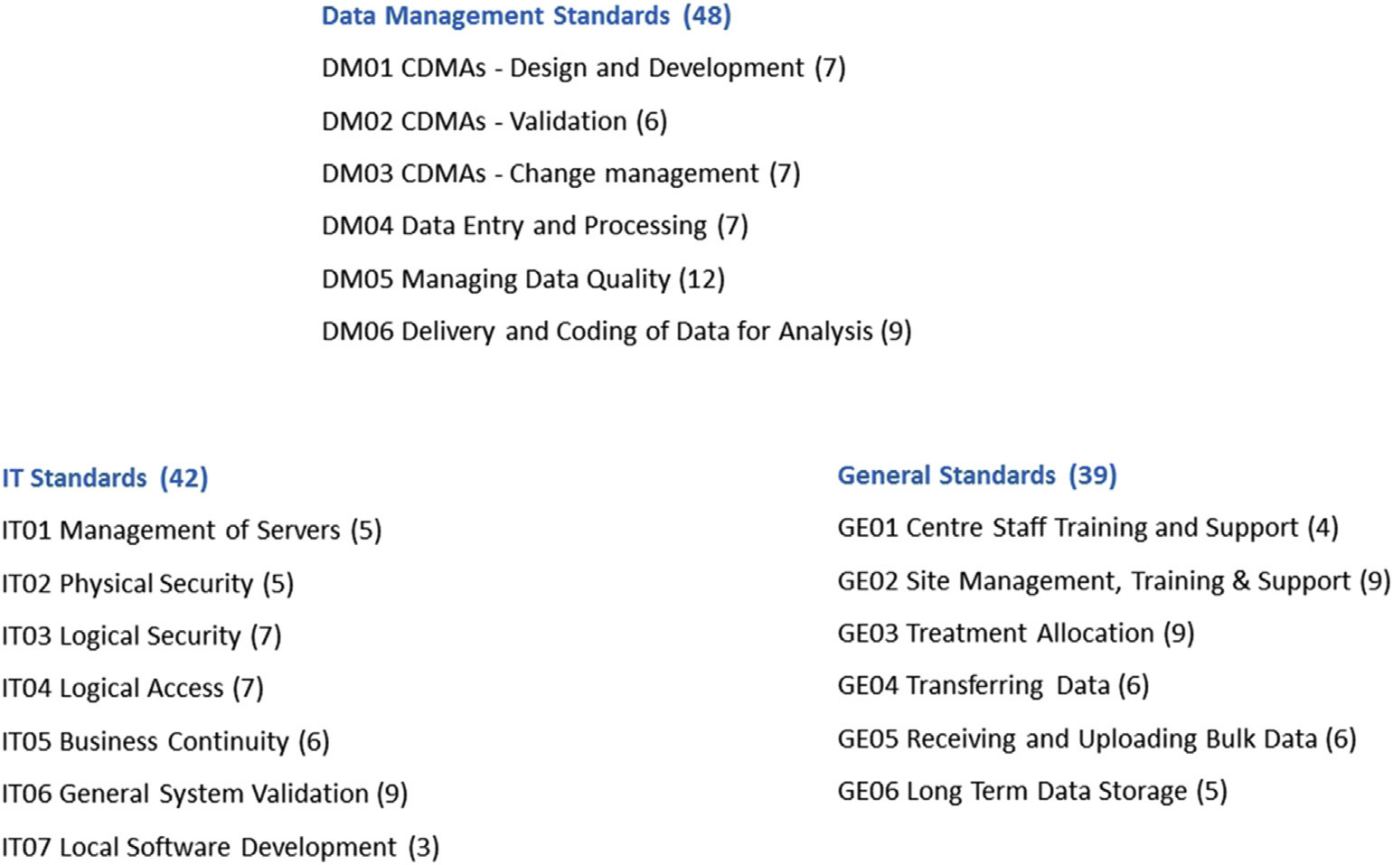
**The current ECRIN standards**. Numbers in brackets refer to the number of standards under each heading. (CDMA = Clinical data management application, the specific database system developed for an individual trial, including the study specific eCRFs, navigation and check logic etc.)

The 129 listed standards, or ‘requirements for data centre certification’, are divided into 19 separate lists, some focused on IT, some mainly concerned with data management, and some that deal with both (see Fig. 1).

In terms of the traditional framework used for quality measures in health care, Donabedian's triad of structure, process and outcome measures [7], the ECRIN requirements are very largely (about 80%) focused on process, with the remainder dealing with structure. Measuring outcome measures was seen as too difficult within a short term audit.

The expectation is that most of the relevant structural components, for instance, a secure network, external firewalls, a clinical data management system (CDMS) that is compliant with GCP, and some form of treatment allocation system will be present. This assumption has been borne out by the audits – the structural components are almost always in place and modern CDMS systems are indeed ‘technically compliant’, for instance they provide audit trails, logical access controls, etc.

But technical compliance is just that – it provides the potential but not the proof of actual compliance. The focus is therefore very much on the quality systems (SOPs etc.), processes, procedures and people that the centre puts around these components to ensure good practice, and – critically – the demonstration of their use in practice.

### 2.3 The process of certification

Yearly (in May) a call for certification of data centres is launched on the ECRIN website with a deadline for application at the end of August. The call is currently restricted to academic trial units from national scientific networks belonging to ECRIN-ERIC members (Germany, France, Italy, Spain, Portugal, Hungary, Norway). The candidates centres are proposed by the national scientific networks in each country.

Certification is based on an on-site audit of the centres' data management and IT services against the standards, carried out over two to three days by a team of auditors, all staff with substantial experience in trials IT, data management or quality assurance. Audits are carried out in English but at least one of the audit team is a native speaker in the centre's working language.

The auditors do not make the certification decision. Instead they produce reports for the ECRIN Independent Certification Board, a body of currently 7 senior professionals in clinical trials management nominated by ECRIN's senior scientific group, the Network Committee. It is the Certification Board that makes the award decision, based on the auditors' reports and any further explanatory information made available by the unit themselves.

The Board also has the discretion to postpone certification, to give a centre the opportunity to correct identified issues, and will then request either documentary evidence of the changes or a later re-audit to verify that the corrective action has been successful. Certified centres are listed on the ECRIN webpage. The costs related to audits (approximately €6000) and the work of the Certification Board are covered by the ECRIN-ERIC budget.

Certification, once awarded, lasts for 4 years. The re-certification process has been designed to be risk-based, so that the extent and duration of any re-audit will be based on a prior assessment of the changes that have occurred within the unit in the intervening years.

### 2.4 The certification programme, to mid-2016

As a result of a pilot phase 2 centres were certified in 2012 (KKS Düsseldorf, Germany; Uppsala Clinical Research Centre, Sweden). Thereafter three regular calls have been launched in 2014, 2015 and 2016 (ongoing), with most of the audits taking place at the end of each year or the beginning of the next.

Six units applied in 2014, of which 5 are now certified (EUCLID, Bordeaux, France; The clinical trial unit, Freiburg, Germany; Mario Negri, Milano, Italy; GIMEMA, Roma, Italy; AIBILI, Coimbra, Portugal) with one certification remaining in progress. In 2015 four applications were received. Three of the units are certified (UPCET, Lyon, France; IZKS Mainz and KKS Marburg, Germany), with certification in progress for the fourth. A summary of the certification programme is given in Table 1.

**Table 1:** Status of certifications for ECRIN data centre certification program.

Call	Applications	Audits	Re-audits	Certification
Pilot	2	2 (in 2011)	2 (in 2012)	2 (in 2012)
2014	6	6 (2 in 2014, 4 2015)	3 (2 in 2015, 1 to do)	5 (4 in 2015, 1 in 2016)
2015	4	4 (4 in 2016)	2 (1 in 2016, 1 to do)	3 (3 in 2016)
2016	4	4 in early 2017	2 in 2017	–

Of the 10 units now certified, only three received certification immediately. The other seven first required some corrective action and either the production of documentary evidence of changes (in 2 cases) or a re-audit (in 5 cases). This indicates that the ECRIN standards are attainable, but certainly not easy, which seems wholly appropriate for standards designed to indicate high quality IT and data management.

## 3 Results

The current small number of ECRIN certified trials units, after just two completed rounds of audits (n = 10) and the still limited number of new multinational clinical trials supported by ECRIN (e.g. 16 in 2016), means evaluation of the impact of the program is confined to relatively local or short acting effects. Nevertheless, it is possible to already identify three areas where such effects are visible. The first is concerned with the development and dissemination of the ECRIN data centre *standards*, the second with the *certification process* itself, and the third is the effect on the *units* that have gone through certification.

### 3.1 Benefits associated with the certification standards

Although IT and data management are services provided to support research, and not ends in themselves, they can cause considerable problems to trial units and even invalidate results if not carried out properly. By raising awareness of key IT/data management issues, and by identifying and promoting good practice, the standards therefore have an important role in the more general drive to reduce wastage in research [8].

The standards also represent the first time that the often vague statements about IT and data management that are found in GCP and other regulatory material have been turned into detailed and pragmatic statements of good practice, tailored to the resources available within not-for-profit trials units. Their perceived usefulness is reflected in some countries by their incorporation into national quality initiatives and documentation:

a) In Switzerland, the Swiss Clinical Trials Organisation developed GCP-compliant guidelines for data management processes in clinical study management closely following the ECRIN standard and incorporated it into their 'Guidelines for Good Clinical Practice' document, making explicit reference to their origin within ECRIN [9].
b) In France, the standards were first translated and then integrated into the “Good Professional Practices” manual for French Clinical Investigation Centres. The translation was published in the journal Thérapie [10].
c) In Germany, initiated by the German clinical trials network (KKSN) and funded by the German Ministry for Education and Research (BMBF) five German trial units were selected by independent reviewers to prepare for the certification as ECRIN data centres. The objective of the grant was to implement the ECRIN requirements in these centres. In fact four German centres have now been certified, and two more have applied as part of the 2016 call.
d) In Asia, a collaboration between ECRIN and the Japanese Academic Research Organisation (ARO) network is currently being explored. As a first step the ECRIN standards (version 3.0) have been translated into Japanese. More details were discussed in a series of workshops (Summer 2016) with training for Japanese auditors arranged for February 2017.

In other countries, for example the UK, the standards have been extensively discussed within the national networks, and some units have decided to use them as definitions of good practice, for instance in the context of self-assessment.

The standards have also been seen as a direct source of expert guidance in clinical trial IT and data management, as evidenced by the steadily rising number of ad hoc queries coming into ECRIN, about the meaning and application of the standards or IT and data management in general. The data management standards have been seen as a useful model for similar initiatives, particularly within other areas of ECRIN. A similar 'standard approach' is currently being considered in nutrition research, in monitoring and in pharmacovigilance.

Reviewing the standards to keep them relevant has helped to raise issues and stimulated debate about ongoing developments in practice. For instance the need to address the increased use of external hosting – e.g. with 'cloud' computing – led ECRIN to initiate a workshop with experts in the field, to try and clarify the risks and benefits associated with such usage [11].

### 3.2 Benefits associated with the certification programme in general

The most obvious benefit has been to identify and/or promote data centres with high quality practice in member countries of ECRIN. This is the prime purpose of the certification programme, and (while the process has only just begun) the certification process does seem to genuinely differentiate and identify high quality IT and data management practice in trials units. Once publicly identified, certified centres can take on a mentor/demonstrator relationship with centres under development. In countries where there already is a mature network of trial units this may be less important (inter-unit relationships can often be competitive rather than co-operative) but in countries with a smaller network of centres having a recognised centre of excellence is seen as a key component in developing that network.

The certification process continually tests the standards in the real world – and ensures that they have real relevance and are not just an academic or static set of suggestions. The audit process is itself a dialog, and the feedback received from the units helps to sustain the review of standards and maintain their relevance. As assessed in a survey, the certification process also raises the knowledge and skills of the auditor group – as one auditor said “By checking (our) own processes against the ECRIN standards you are able to find deficiencies and by reading the Explanation and Elaboration you are able to find possible solutions”, and several auditors have made similar remarks. The auditors are experienced and often influential staff, so this not only helps to raise general awareness of good IT and data management but also helps to disseminate it through the wider clinical trial community.

A process of recognition of the programme by regulatory bodies has begun. The programme has been presented to the EMA GCP Inspectors Working Group (December 2015) and to a representative of the German Expert Group of Inspectors dealing with Computer based Systems (August 2015).

### 3.3 Benefits for the trial units certified

The audited units have all given positive feedback about the audit process itself. Although they need to prepare for the audit they receive in return (effectively) 2–3 days of free consultancy from experienced staff. Even the best centres can obtain benefit from inspection of their processes and resulting discussion, and we know, from feedback received indirectly, if informally, through the national networks, the centres themselves confirm this. The audits often accelerate developments already planned or being considered, sometimes making it easier for resources to be found, for example from a parent organisation. There is rarely a disagreement on the auditors' findings of non-compliance – more normally the unit agrees there is a deficiency but explains that they have not yet had a chance to deal with it. The certification process itself can be a powerful incentive to get these issues resolved.

Successful certification brings with it affiliate membership of ECRIN, through a framework contract with the certified data centre. This in turn makes it much easier to include the unit (as a linked third party) in Horizon 2020 bids and other funding initiatives where ECRIN is a partner. Until recently, this has been of relatively limited importance, partly because certification has been limited to only a few units and partly because the framework contracts only recently started to be implemented. As a consequence, certified units have only been involved in a few EU FP7/H2020-funded projects so far (6 ECRIN studies supported by 3 certified data centres), but the hope and expectation is that this will increase in the coming years due the framework contracts between ECRIN and the certified data centres.

## 4 Discussion

The audit based certification mechanism used by ECRIN is one of several approaches and initiatives that have been taken to improve quality within non-commercial clinical trials units. Each European country has its own official regulatory and inspection regime of course, but anecdotal evidence indicates that the time and resources these have available to examine academic research units (if they are not Phase I units) is highly variable. In some countries inspection of such units occurs regularly, in others as necessary, on the basis of a risk assessment, and in others it seems to hardly occur at all.

### 4.1 Promoting quality through training

One approach to promoting quality focuses on training and supervision of staff. Several organisations offer training courses with certification for individual professionals in clinical data management. Examples are the Society for Clinical Data Management, a non-profit, international organisation, which established the Certified Clinical Data Manager program for clinical data managers [12], and Barnett International, a for-profit organisation in clinical research training, that provides a 30-Hour Clinical Data Management On-Boarding Program with certification [13].

The ECRIN standards include requirements that policies for initial and continuing training (of IT and data management staff) are in place, and that training should be clearly recorded and regularly reviewed on an individual basis. They do not however, stipulate any particular training course for any role, recognising the very wide range of provision that is available, including locally created courses and support (though there is an expectation that fundamental issues like GCP and data protection are covered, by both initial and continuing training programmes). The proper preparation of personnel is a prerequisite for GCP compliance, so the focus is on the unit demonstrating that it can identify training needs, manage the training processes that follow, and maintain the required levels of competence. Specific training programmes are seen as complementary to the more general ECRIN requirements.

### 4.2 Registration

Another approach to identifying quality is by using self-assessment and self-reporting, against a pre-defined set of criteria. This is the method used in the UK, within its registration scheme for non-commercial clinical trial units. Registration brings both reputational and financial rewards, so the trials units are highly motivated to seek registered status. Within the registration process, an international review panel assesses compliance with the stipulated competencies every 5 years. In a recent report, improvements in IT systems and hosting of clinical trial databases were claimed as a result of the registration process [14]. ECRIN considered a similar methodology for promoting quality but decided that the reliance on self-reporting, though a good initial step and certainly cheaper to implement than an audit based system, would give insufficient credibility to the certification decision.

### 4.3 Other audit based systems

In other countries, external audits are used. In Germany, for example, the membership of an academic trial unit in the national scientific network (Network of the Koordinierungszentren für Klinische Studien (KKSN)) is based on a successful external audit, that includes DM and IT procedures [15].

A voluntary certification scheme for commercial and non-commercial phase I units was created by the MHRA in the UK as a consequence of the TGN1412 incident, in which several healthy volunteers in a Phase I study in 2006 suffered multiple organ failure [16]. Version 3 of their requirements for accreditation was published in October 2015, though the focus is very much, as one would expect, on patient safety. The scheme is based on successful inspection against the 20 listed requirements.

The British Standards Institution (BSI) and the Alliance for Clinical Research Excellence and Safety (ACRES), in the US, announced in early 2016 an initiative to develop global standards of excellence for clinical research sites (rather than trials units) [17]. The intention is to create “a comprehensive set of standards covering … patient and subject engagement and protection, site personnel, research integrity, facilities, information systems and data management, management and administration as well as quality management”.

According to the web site: “These standards and derived metrics will enable an innovative ‘dynamic accreditation’ process that automatically monitors operational data in real time to continuously improve site and system performance, benefitting stakeholders while minimizing research site burdens.” It is unclear what this will mean in practice but the approach is an interesting one and could be very relevant to the ECRIN certification programme in the future, especially if that extends beyond IT and data management to other areas (e.g. pharmacovigilance, monitoring).

### 4.4 Using ISO standards

Formal certification, e.g. using ISO standards, is another approach (and several trials units have or are investigating certification of their quality systems with ISO 9001). Within the ECRIN certification scheme we examined the ISO 27000 series in particular and borrowed some concepts from ISO 27001 (dealing with IT security management), for instance the need for an ongoing security management system [18].

Although we acknowledge the importance and usefulness of the ISO 27000 family of standards, we decided that they were not always well adapted to relatively small organisations such as academic trial units, and felt that the lack of freely available supporting tools and services would also be a problem for academic units trying to develop compliance with the ISO standards. In addition a decision to seek ISO certification, or not, would most often be taken at an institutional level and be outside the control of a trials unit. We therefore did not incorporate ISO certification, in any form, into the ECRIN standards.

We have also discussed developing the ECRIN certification process into an ISO or similar formal scheme. This offers the potential of increased credibility but – certainly to begin with – the additional costs and administrative overhead incurred (including the costs of having ECRIN accredited as an ISO certifying organisation), and the increased costs of carrying out each unit certification for ECRIN, were seen as prohibitive. It may be, however, that after some years' operating the certification scheme, this question could be usefully re-examined.

### 4.5 The ECRIN approach – audits against public standards

In the end ECRIN decided to choose a ‘middle way’ and establish an audit based certification scheme using its own standards. We believe this has greater credibility than a system based solely on self-assessment, but it avoids the substantial costs of working against ISO standards.

By using public, published standards the certification programme is far more transparent and understandable than a scheme based upon the private thoughts and concerns of individual 'expert' auditors. We believe this approach also makes the audit process much less intimidating and more productive. By using continuous review of public standards the programme not only ensures the ongoing relevance of those standards, it also helps to characterise and disseminate good practice within the clinical trials community generally, and to identify new issues that are appearing in practice, where trials units are often looking for information and support.

### 4.6 Future plans

Having said that, it is true that we are suffering from a 'critical mass' effect. Certification needs to be widespread enough for the trials community as a whole to understand it, to see that it has been accepted as valid by key stakeholders, and for the benefits of certification to be clear. The certification programme has not yet reached that critical mass. The key will be to continue to build up the numbers of certified units and at the same time continue to develop, and publicise, the strengths and positive effects of the programme [19].

Exactly how many certified data centres are needed is a question that has been debated often within ECRIN but as yet is not fully resolved. One viewpoint is that only a limited number of certified centres are necessary to actually support ECRIN services in multinational clinical trials. This argument is based on the number of ECRIN supported studies and the number of requests for data management services. The alternative view is that certification should be widespread, as part of the drive to increase the capacity and quality of clinical research in Europe on a wider scale.

It may be that a single pan-European approach is not possible, because of different strategies and available resources in different member states. For example, countries with many large developed units (e.g. Germany) may prefer to qualify a substantial number of data centres, whereas those with fewer and/or newer research centres (e.g. Portugal) might want only a few dedicated centres to be certified, with these then providing data management services to other clinical trial units in their country. In some countries, where data management is currently outsourced to commercial companies, we may need to consider making certification available to CROs.

Currently, the programme is restricted to ECRIN ERIC member countries and financed by the ECRIN ERIC budget. To broaden the certification programme and to increase awareness and acceptance by the scientific community, the plan is to make certification available to ECRIN-ERIC ‘observer’ countries in 2017, although a not-for-profit charge will be made. Currently ECRIN is planning further meetings and discussions to establish the next steps for the certification programme, and to clarify the best ways in which ECRIN can continue to promote quality in clinical research.

## List of abbreviations

ACRES: Alliance for Clinical Research Excellence and Safety
AIBILI: Association for Innovation and Biomedical Research on Light and Image (Coimbra, Portugal)
ARO: Academic Research Organisation
BMBF: Bundesministerium für Bildung und Forschung
BSI: British Standards Institute
CDMA: Clinical Data Management Application (the study specific database)
CDMS: Clinical Data Management System (the underlying software system)
CRO: Contract Research Organisation
DM: Data management
eCRF: electronic Case Report Form
ECRIN: European Clinical Research Infrastructure Network
ECRIN-NC: ECRIN Network Committee
EMA: European Medicines Agency
ERIC: European Research Infrastructure Consortium
EU: European Union
EUCLID: Academic EUropean CLInical Trials Services Platform & Development (Bordeaux)
FP7: Framework Programme 7
GCP: Good Clinical Practice
GIMEMA: Gruppo Italiano Malattie EMatologiche dell'Adulto
H2020: Horizon 2020
ICB: Independent Certification Board
ISO: International Organization for Standardization
IT: Information Technology
IZKS: Interdisziplinäres Zentrum Klinische Studien (Mainz)
KKS: Koordinierungszentrum für Klinische Studien
KKSN: Network of the Koordinierungszentren für Klinische Studien
SOP: Standard Operating Procedure
UPCET: Unité de Pharmacologie Clinique et Essais Thérapeutiques (Lyon)
